# Recurrent episodes of micturition with expulsion of symphyseal plate screws following pelvic ring fixation: case report

**DOI:** 10.1186/s12891-015-0581-7

**Published:** 2015-05-28

**Authors:** Eli Peled, Doron Norman, David Kakiashvili, M. Bradford Henley

**Affiliations:** Department of Orthopedic Surgery B, Rambam Health Care Campus, P.O. Box 9602, Haifa, 31096 Israel; Department of Urology, Rambam Health Care Campus, Haifa, Israel; Department of Orthopaedics and Sports Medicine, University of Washington, Seattle, WA USA

**Keywords:** Symphyseal plate screws, Traumatic symphyseal disruption, Urinary expulsion

## Abstract

**Background:**

Based on a computer-assisted literature search, this case is the first description of repeated loosening of metallic internal fixation implants after pelvic ring stabilization, associated with intravesical metal migration and micturition with expulsion of two bone screws.

**Case presentation:**

A 62-year old woman was seen after the urinary expulsion of a 6.5 mm diameter cancellous screw. About seven years earlier, she had been hit by a motorcyclist while crossing the street. On admission at the time of the initial injury, thoraco-abdominal computerized tomography with intravenous contrast material revealed a bladder injury and pelvic ring fractures. An anterior-posterior type injury to the pelvic ring was diagnosed with symphyseal pubis disruption, and widening of the left sacroiliac joint with an associated sacral fracture. Explorative laparotomy revealed two bladder lacerations of both the posterior and the anterior bladder wall, which were repaired primarily. Orthopedic surgeons reduced the pelvis and stabilized it with two plates and screws. Seven years after the original injury, the patient presented with recurrent abdominal pain after expelling a screw into the toilet while urinating. Planar radiographs showed only five of the original screws remaining in the two symphyseal plates, and all screws appeared to have loosened when compared to the original fixation radiograph.

**Conclusion:**

This clinical report emphasizes the importance of symphyseal plate positioning and the sequelae of imprecise positioning, especially postero-superiorly adjacent to the Retzius space. The presence of protruding metal prominences, even smooth ones like a plate corner or screw head, might endanger the bladder. When using superior plates, imprecise contouring may lead to plate edge protrusion which could damage the bladder even long after application.

**Electronic supplementary material:**

The online version of this article (doi:10.1186/s12891-015-0581-7) contains supplementary material, which is available to authorized users.

## Background

Pelvic ring injuries with traumatic symphyseal disruption are associated with acute bladder rupture in up to 5-6 % of cases [1, 2]. In general, loosening of fixation after orthopedic repairs may occur when a fixation implant crosses a joint, or when an implant is applied to osteoporotic bone or is applied in a non-rigid fashion allowing micromotion. Bladder rupture after open reduction with internal fixation of the pelvic ring has been described previously as a late event in two cases [3, 4]. Acute bladder entrapment by external fixation has also been described [5].

## Case presentation

**Fig. 1:**
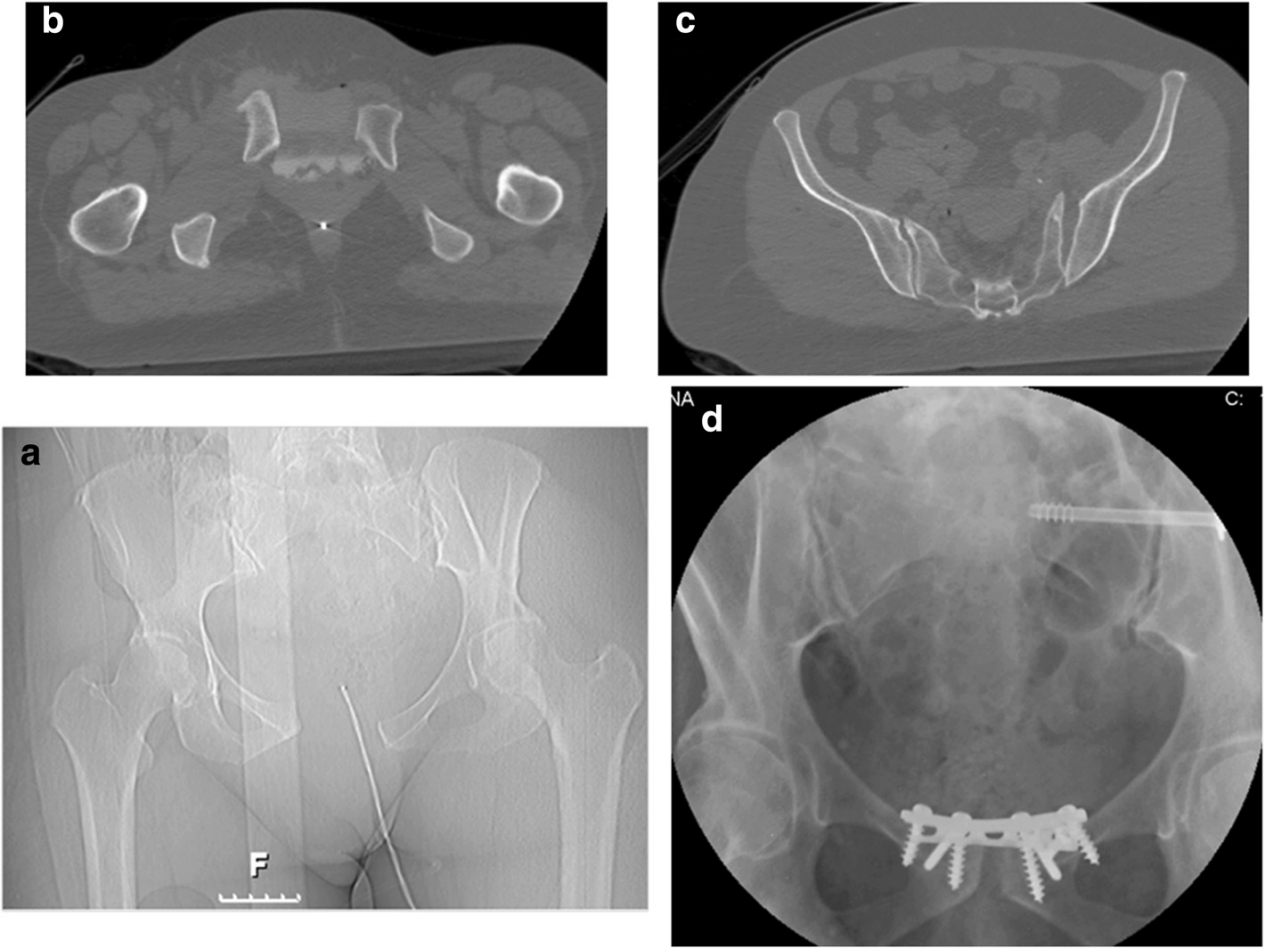
CT scan images showing the acute pelvic ring injury and AP pelvis post-operative x-ray. CT scan images of initial injury (November 2002). Scout image [**a**] shows the AP pelvic ring injury with diastasis of the symphysis pubis and left sacroiliac joint dislocation; [**b**] Axial image at the level of the symphysis pubis, showing diastasis of the symphysis pubis associated with extravasation of the contrast material from the bladder, indicating bladder injury; [**c**] Axial image at the level of the sacroiliac joint, showing widening and posterior dislocation of the left sacroiliac joint with rupture of the anterior sacroiliac ligaments; [**d**] AP pelvis image taken about six weeks post injury shows fixation of the symphysis pubis by two plates spanning the symphysis pubis and additional left iliosacral S1 screw. The symphysis is stabilized by seven screws; four 6.5 mm diameter cancellous screws and three 4.5 mm diameter cortical screws. The iliosacral screw is a 7.3 mm diameter cannulated screw with a washer flush to the outer table of the ilium, indicating compression between the screw and the bone

A 62-year old woman was admitted to our Emergency Department after the urinary expulsion of a 6.5 mm diameter cancellous screw. She reported low abdominal pain and recurrent urinary tract infections during the preceding five months. About seven years earlier, she had been involved in a motor vehicle crash as a pedestrian, being hit by a motorcyclist while crossing the street. At the time of this initial injury seven years ago when admitted to the Emergency Department, she was conscious with a GCS of 15, complained of low abdominal pain and an open laceration of the left calf. On initial survey, FAST was normal and macroscopic hematuria was noted. Thoraco-abdominal computerized tomography (CT) with intravenous contrast material revealed a bladder injury and pelvic ring fractures. There was both intraperitoneal and extraperitoneal extravasation of the contrast material from the bladder into the space of Retzius. An anterior–posterior type injury to the pelvic ring was diagnosed with symphyseal pubis disruption, and widening of the left sacroiliac joint with an associated sacral fracture (Fig. 1). An explorative laparotomy confirmed the bladder laceration. There were two lacerations, one of the posterior bladder wall and a larger one involving the anterior bladder wall. The bladder was repaired primarily and the urologists protected the repairs with both urethral and suprapubic catheters. Orthopedic surgeons reduced the pelvis and stabilized it with two plates and screws; a postero-superior 4-hole, 4.5 mm dynamic compression plate (DCP) with two 6.5 mm cancellous screws on each side of the symphysis pubis and an additional antero-superior 5-hole, 3.5 mm DCP with three 4.5 mm cortical screws. An additional left-side 7.3 mm diameter x 65 mm length cannulated cancellous iliosacral screw with a washer at the level of S1 (Fig. 1) were placed. Two postoperative cystograms confirmed the absence of a bladder leak. The urinary catheters were removed successively, the suprapubic initially at three weeks postoperatively. Another cystogram performed about two months later was normal.

**Fig. 2:**
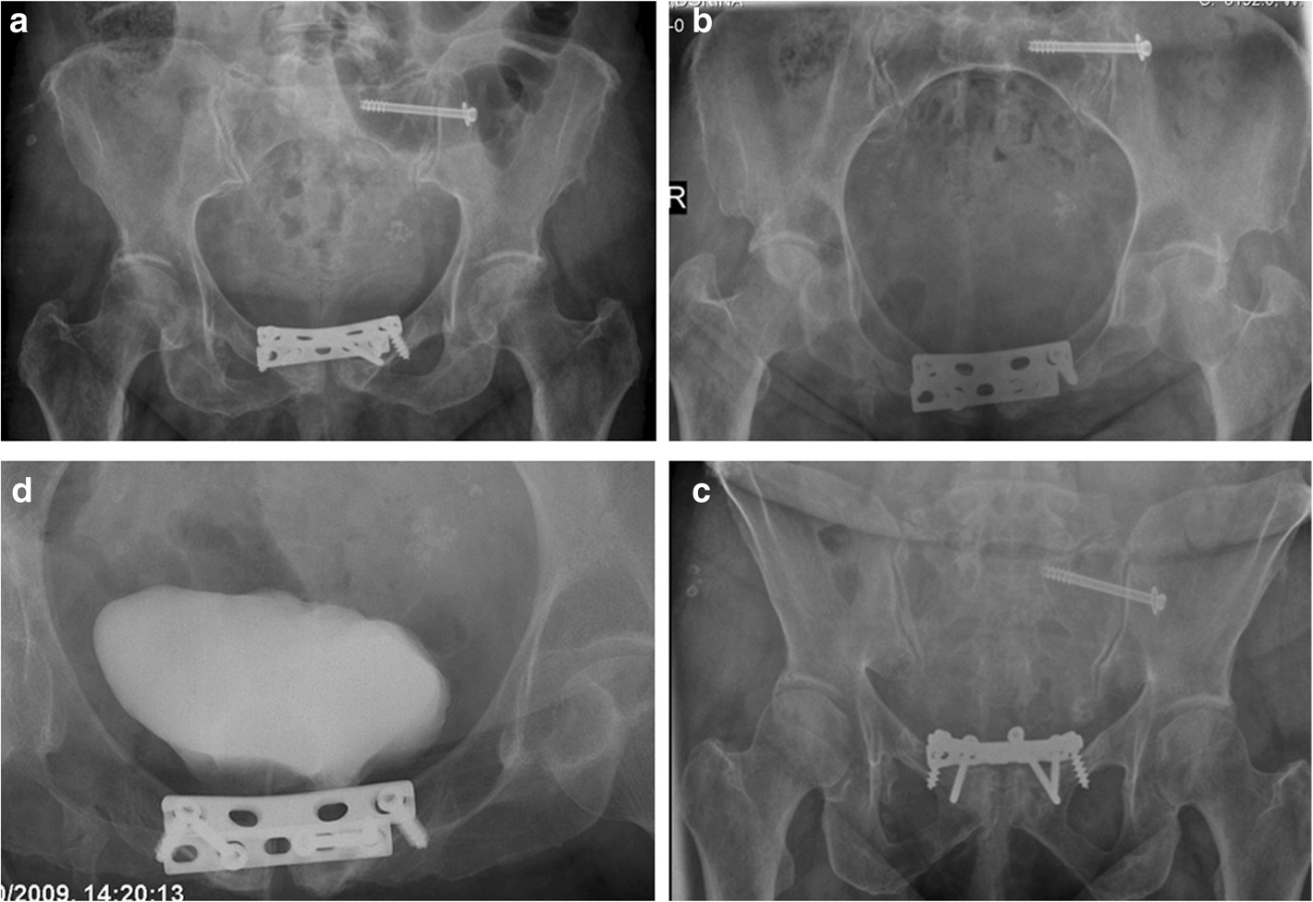
Pelvic ring x-rays and a cystogram after repeated screw expulsion. Pelvic ring images AP [**a**] inlet [**b**] and outlet [**c**] views, obtained after the expulsion of the second screw (October 2009). The three projections show five screws remaining compared to the seven screws which had been placed initially (compare with Fig. 2). Two 6.5 mm cancellous screws are present. All five remaining screws have loosened as seen in the three views. The position of the washer on the S1 iliosacral screw has changed and is no longer flush to the outer iliac cortex. These changes indicate that the 7.3 mm screw has also loosened when compared to its original position. A cystogram view [**d**] shows the contrast material adjacent to the posterior 4.5 mm DCPlate with its two 6.5 cancellous screws

**Fig. 3:**
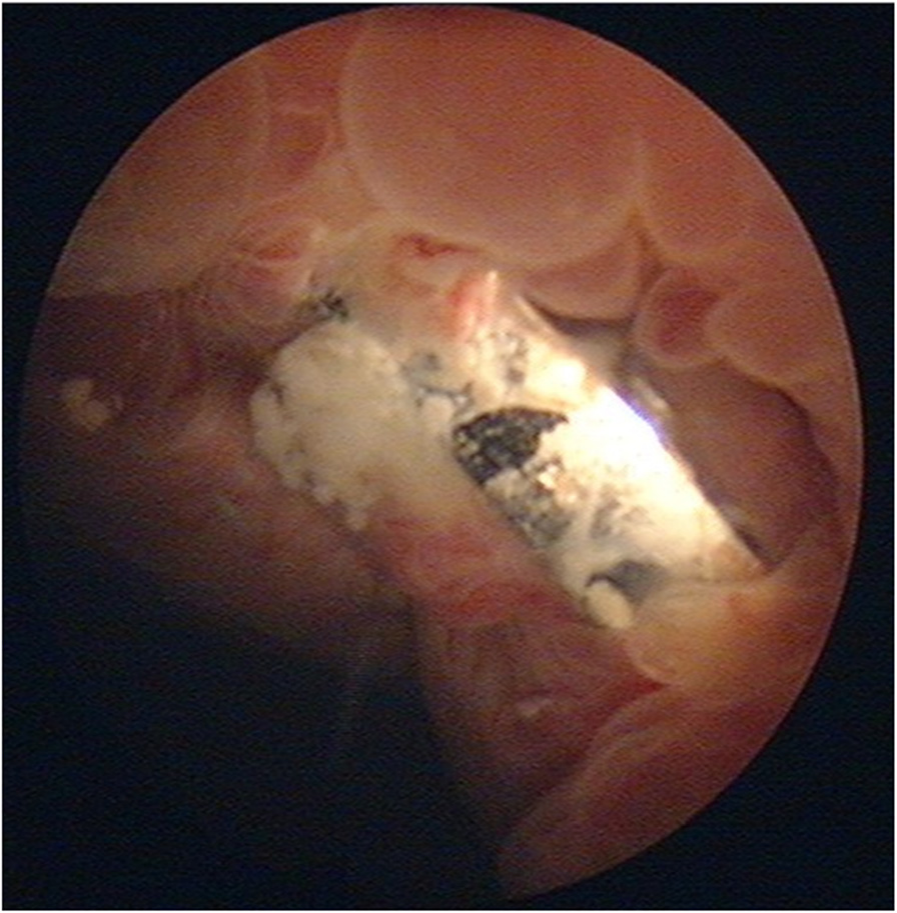
Cystoscopy image with bladder perforation. Cystoscopy performed prior to plate removal and bladder repair showing perforation of the anterior bladder and intrusion of the posterior 4.5 mm DCPlate

Seven years after the original injury, the patient presented to our Emergency Department with recurrent abdominal pain, after she had expelled a screw into the toilet while urinating. During the previous five months she had recurrent urinary tract infections. Planar radiographs were obtained and showed only five screws remaining in the two symphyseal plates. All screws appeared to have loosened (Fig. 2) when compared to the original fixation radiograph. CT scan and cystogram showed no bladder foreign bodies. However, the cystogram demonstrated 1.5-2 cm diameter bladder ulceration in the anterior wall, with the posterior pubic plate being seen through the laceration (Fig. 3). Urine culture was positive with a mixture of Staphylococcus aureus and Escherichia coli, and the patient was treated according to their antibiotic sensitivities. During her hospitalization, the patient mentioned another episode of a screw being expelled while urinating three years earlier (four years after the accident). After that episode, she reported improvement and disappearance of the low abdominal pain, without consulting a physician.

**Fig. 4:**
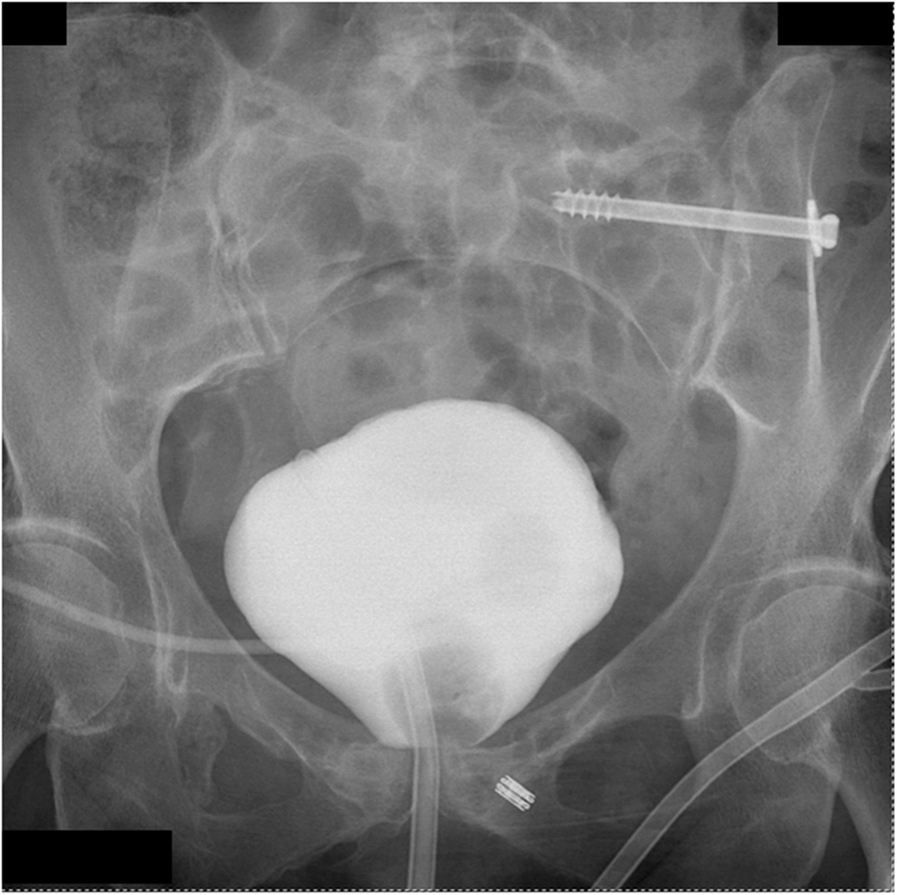
Cystography after hardware removal and bladder repair. AP pelvic x-ray taken during cystography after symphyseal hardware removal and bladder repair shows no bladder leak. The cystoscopy shows the two urinary catheters used during the operative repair

Following eradication of the bladder infection, the patient had elective surgery for removal of the symphyseal plates and repair of the bladder ulceration. The previous Pfannsetiel incision was used for exploration. A pressure ulceration of the anterior bladder wall was contiguous with the posterior-superior plate which was protruding into the bladder and associated with multiple adhesions between the outer bladder wall, the pubic bones and the Retzius space. The plates were removed and the bladder was repaired primarily in a double layer, protecting the repair again with both suprapubic and urethral catheters. Bone cultures taken during the procedure showed growth of no organisms. A cystogram two weeks later was normal without leak and the catheters were removed (Fig. 4). Two months post removal, follow-up was normal and the patient denied any additional urinary symptoms and had sterile urinary cultures.

## Discussion

Urinary tract infections, especially cystitis, are common. One of the reasons for recurrent urinary tract infections is foreign bodies. Intravesical migration of a screw associated with symptoms of a urinary tract infection is uncommon. Fridman et al. described a patient who voided a screw after undergoing pelvic fixation years earlier [3]. The patient was investigated by cystoscopy which revealed that a washer had migrated into the bladder. The washer was removed and the patient was treated by urethral catheter without further urinary tract complications. Dietrick et al. [6] described an intrauterine device migration into the bladder many years after insertion. In these circumstances, the devices are usually surrounded by calcifications and associated with recurrent urinary tract infections.

Symphysis pubis internal fixation is usually performed with a single plate positioned superiorly and fixed to the left and right superior pubic rami [7, 8]. In cases which necessitate more stability, an additional plate is used, with the second plate being placed anteriorly [9]. Our patient had fixation with two plates, the first plate placed superiorly, and the second placed behind the symphysis pubis facing the Retzius space. Over the years, her screws loosened, most probably due to repetitive motion. During this process, the screw heads likely protruded posteriorly into the Retzius space, eroding and perforating the bladder wall. The first screw migrated into the bladder and was voided without clinical sequel. The episode described here occurred a few years later when a second screw had loosened, then migrated into the bladder causing recurrent cystitis until it was voided spontaneously.

## Conclusion

This clinical report emphasizes the importance of symphyseal plate positioning and the sequelae of imprecise positioning, especially postero-superiorly adjacent to the Retzius space. The presence of protruding metal prominences, even smooth ones like a plate corner or screw head, might endanger the bladder. When using superior plates, imprecise contouring may lead to plate edge protrusion which could damage the bladder even long after application.

### Consent

Written informed consent was obtained from the patient for publication of this Case Report and any accompanying images. A copy of the written consent is available for review by the Editor of this Journal.

## Abbreviations

GCS: Glasgow coma scale
FAST: Focused assessment with sonography in trauma
CT: Computerized tomography
DCP: Dynamic compression plate
